# Atomic Force Microscopy Images Label-Free, Drug Encapsulated Nanoparticles In Vivo and Detects Difference in Tissue Mechanical Properties of Treated and Untreated: A Tip for Nanotoxicology

**DOI:** 10.1371/journal.pone.0064490

**Published:** 2013-05-28

**Authors:** Dimitrios A. Lamprou, Vinod Venkatpurwar, M. N. V. Ravi Kumar

**Affiliations:** Strathclyde Institute of Pharmacy and Biomedical Sciences, University of Strathclyde, Glasgow, United Kingdom; Dalhousie University, Canada

## Abstract

Overcoming the intractable challenge of imaging of label-free, drug encapsulated nanoparticles in tissues *in vivo* would directly address associated regulatory concerns over 'nanotoxicology'. Here we demonstrate the utility of Atomic Force Microscopy (AFM) for visualising label-free, drug encapsulated polyester particles of ∼280 nm distributed within tissues following their intravenous or *peroral* administration to rodents. A surprising phenomenon, in which the tissues' mechanical stiffness was directly measured (also by AFM) and related to the number of embedded nanoparticles, was utilised to generate quantitative data sets for nanoparticles localisation. By coupling the normal determination of a drug's pharmacokinetics/pharmacodynamics with post-sacrifice measurement of nanoparticle localisation and number, we present for the first time an experimental design in which a single *in vivo* study relates the PK/PD of a nanomedicine to its toxicokinetics.

## Introduction

Nanomedicines are multiple component systems whose distribution *in vivo*, targeted or otherwise, remains a critical area of understanding the clinical outcomes. [1], [2] Apparently small changes in size, shape and surface properties influence their bio-distribution.[3]–[6] Understanding the bio-distribution involves use of labelled particles that rely on fluorescence, luminescence, optical or radioactivity [7]–[9] or approaches that monitor the molecular vibrations. [10] However, labelled nanoparticles cannot be guaranteed to model the behaviour of label-free nanoparticles encapsulating a drug. [11], [12] Nor can spectral imaging techniques describe the micromechanical environment of tissue in which nanoparticles reside. This uncertainty hampers progress in evaluating the therapeutic efficacy and safety of nanomedicines, [13], [14] which ideally should be obtained during the same *in vivo* experiment used to determine the nanoparticles’ tissue distribution and environment.

In order to image the label-free, drug-encapsulated nanoparticles (NPs) in the tissues, and measure the local influence of a particle on the tissue mechanical properties, we adapted *ex vivo* and *in vivo* imaging approaches using an AFM. *Ex vivo* experiments involved the isolation of rat kidney and blood followed by their exposure to an appropriate volume of cyclosporine (CsA) containing NPs. *In vivo* experiments involved the dosing of rats with CsA-NPs either by intravenous (*iv*) or *peroral* (*po*) routes, followed by tissue isolation, sectioning and imaging/measurement of the QNM properties (such as Young’s Modulus, Ε/Pa). Support for the requirement to measure the QNM of tissues during disease progression comes from a significant body of literature: i) the stiffness of red blood cells (RBCs) plays a major role in whole blood viscosity that is correlated to several cardiovascular diseases; [15] ii) the stiffness of tissues such as liver has been established as an independent predictor of liver failure, hepatocellular carcinoma and mortality in cirrhotic patients; [16] iii) arterial wall stiffening, stiffness of carotid artery and aorta is believed to increase in diabetes and can serve as predictors for cardiovascular mortality in end-stage renal disease; [17] iv) the change in cancer/tumor cell stiffness affects the way these cells spread [18], and very recently AFM has been used to study the stiffness of breast cancer tissues from patients. [19] Thus, the AFM is a multifunctional toolbox for the study of the nano-bio-interface, facilitating a better understanding of the pathology and toxicology with resolution down to 0.1 nm has made an essential tool for imaging and measure the mechanical properties. [20] By putting the AFM toolbox in the context of drug delivery, this study presents new possibilities by which we can better address regulatory requirements for nanomedicines, and understand their efficiency and biophysical interactions of particles with tissue, which will be critical in developing effective drug carriers. Here we address the goal of imaging of label-free drug-encapsulated nanoparticles distributed in various tissues *in vivo* and their concomitant quantitative nano-mechanical (QNM) properties after intravenous or *peroral* administration using Atomic Force Microscopy (AFM).

## Materials and Methods

### Materials

Poly(lactide-co-glycolic) acid (PLGA) (Resomer R503H; MW 35–40 kDa) was purchased from Boehringer Ingelheim, (Ingelheim, Germany). Polyvinyl alcohol (PVA) (MW 30–70 kDa) and ethyl acetate were purchased from Sigma-Aldrich (Poole, UK). Cyclosporine (CsA) was purchased from Fluorochem Ltd. Derbyshire, UK.

### Preparation and Characterization of CsA Encapsulated PLGA Nanoparticles

The CsA-NPs were prepared by the emulsion–diffusion–evaporation method. PLGA (50 mg) and CsA (7.5 mg) were dissolved in ethyl acetate (2.5 ml) under stirring at 1000 rpm over a period of 2 h. This drug containing polymer solution was added in drop wise manner to 5 ml of PVA solution (1% w/v). The resulting primary emulsion (o/w) was stirred over 1 h at 1000 rpm followed by homogenization at 15,600 rpm for 15 min to reduce the droplet size. The emulsion was transferred to 25 ml of water and stirred overnight to facilitate diffusion of organic solvent and evaporation. The drug entrapment efficiency was measured by HPLC following previously developed method in our laboratory; [21] the particle size was measured using zeta sizer, nanoSight (*Detailed methods of characterisation and data in the Supplement Information Figures S1 & S2*).

### AFM Measurements

AFM images were obtained by scanning the mica surface in air under ambient conditions using AFM (Digital Instruments, Santa Barbara, CA, USA; Bruker Nanoscope analysis software Version 1.40) operated using the new PeakForce QNM mode. The AFM measurements were obtained using ScanAsyst-air probes, and the spring constant (Nominal 0.4 N/m) and deflection sensitivity has been calibrated, but not the tip radius (the nominal value has been used; 2 nm). AFM images were collected from two different samples and at random spot surface sampling (at least five areas per sample). The quantitative mechanical data was obtained by measuring DMT modulus/Pa using Bruker software (NanoScope Analysis) (*Supplement Information Figure S3*). To obtain the Young’s Modulus, the retract curve is fit using the Derjaguin-Muller-Toporov model, for that reason called DMT Modulus [22].

### Ex vivo Studies with Tissues

To demonstrate the proof of concept firstly we have performed *ex vivo* studies. The *ex vivo* studies were performed using rat blood, plasma and serum (900 µl) to which 100 µl of CsA-NPs were added and vortexed for 1 min and 5 µl of the respective samples were mounted on mica followed by air drying over 10–15 min for AFM analysis as described above. Further we have also used freshly excised kidney from rat to which 50 µl of CsA-NPs were injected using insulin syringe (25G needle). The particle injected kidney was stored at −20°C over 48 h and then cut into 15 µm sections using cryotome and mounted on mica and AFM analysis was performed as described. The kidney without CsA-NPs also processed under same conditions served as a control. To ascertain the role of particle concentration on the tissue stiffness, freshly cut sections of kidney and liver were mounted on mica and 5 µl of CsA-NPs were placed on the sections and allowed to dry for 30 min and analysed by AFM as described.

### In vivo Studies in Sprague Dawley (SD) Rats

All animal experiments included in this study were part of ongoing studies performed under UK Home Office project licence and had received ethical clearance from the University of Strathclyde Ethics Review Panel. SD rats weighing 200–250 g were used in these studies. *Part 1:* CsA-NPs (15 mg/Kg) were administered to male SD rats through tail vein and sacrificed 15 min post injection. Blood was withdrawn by cardiac puncture into an EDTA coated tube and centrifuged at 3000 rpm for 10 min at 5°C for plasma and serum separation. The organs were collected and stored at −20°C until AFM analysis. For AFM analysis, 5 µl of blood, plasma and serum were mounted on mica, separately, while 15 µm sections of kidney, liver and brain were cut using a cryotome and mounted on mica and imaged as described above. *Part 2:* CsA-NPs (15 mg/Kg) were administered to male SD rats by *po* route using a gavage needle. The blood was withdrawn at 2, 4 and 6 h via the tail vein and tissues harvested and processed as above.

### Statistical Analysis

The stiffness data were analysed using Student unpaired t test (Graph pad software). A value of *p*<0.05 was considered statistically significant and *p*>0.05 considered insignificant.

## Results, Discussion and Conclusions

### CsA Particle Characteristics

**Figure 1:**
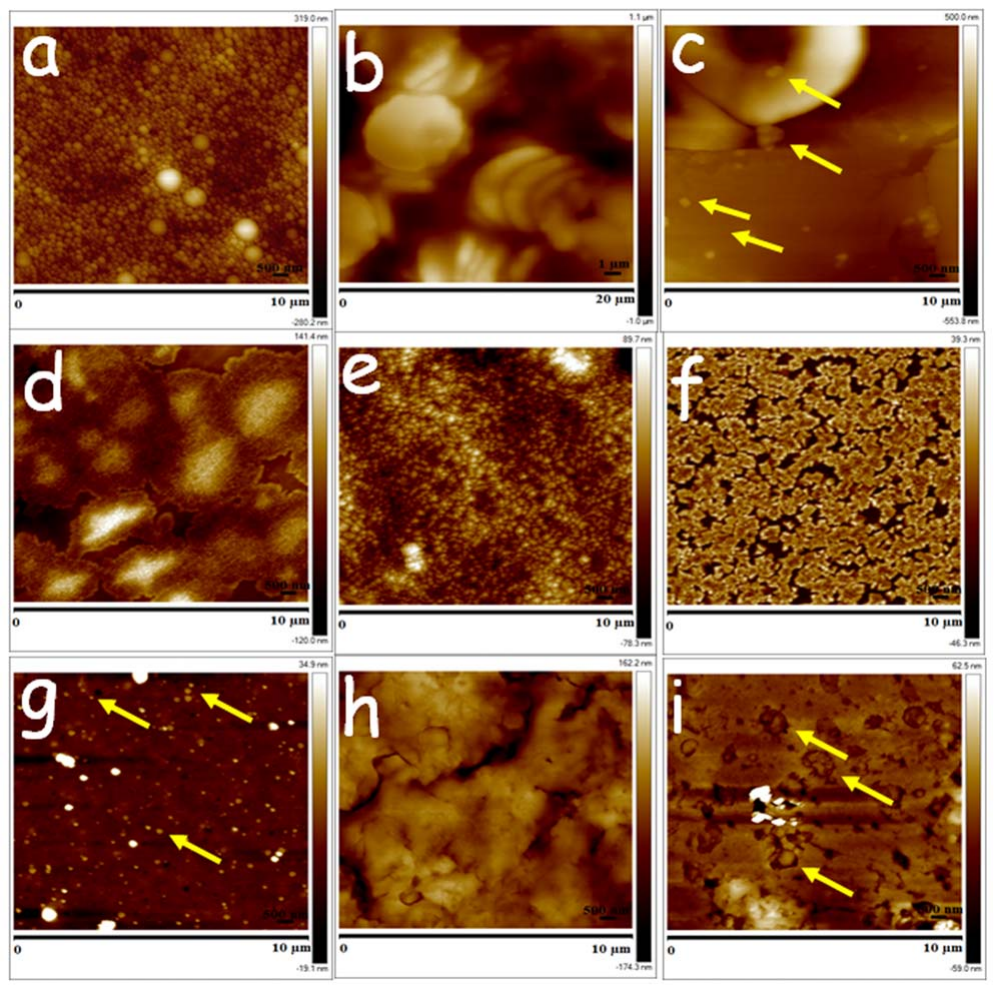
*Ex vivo* samples demonstrating proof of concept that AFM is able to visualise label-free drug-encapsulated NPs in tissues (a) CsA-NPs, (b) blood, (c) blood+NPs, (d) plasma, (e) plasma+NPs, (f) serum (g), serum+NPs, (h) kidney and (i) kidney+NPs. (representative nanoparticles are marked by yellow arrows).

**Figure 2:**
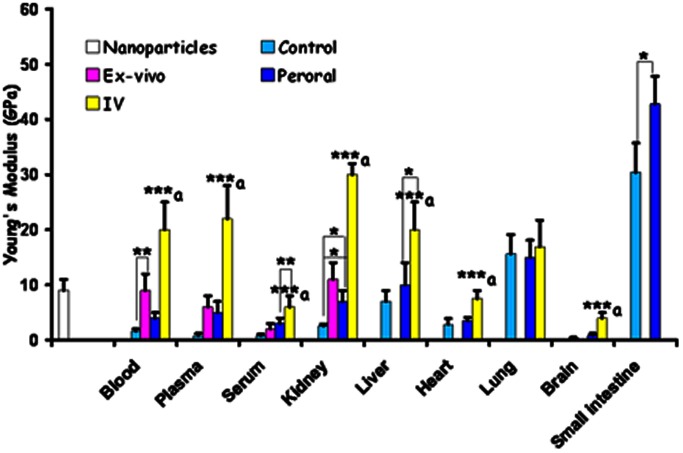
Quantitative nano-mechanical properties of nanoparticles only and various *ex vivo*/*in vivo* tissues untreated or treated. Tissue stiffness is measured as Young’s Modulus (GPa) *p<0.05; **p<0.01; ***p<0.001 a *vs* control, *ex vivo* & oral (not applicable to serum, liver & kidney).

The particle sizes were 280±15 nm (by zeta sizer) and 254±72 nm (nanoSight) (Supplement Information Figures S1& S2). The AFM imaging of the particles revealed spherical shape particles with average size was ∼280 nm with few larger particles of ∼665 nm (Figure 1 a) and had a stiffness of 9±2 GPa (Figure 2). The drug entrapment efficiency was ∼60% at 15% CsA w/w of polymer (50 mg).

### Ex vivo Studies

**Figure 3:**
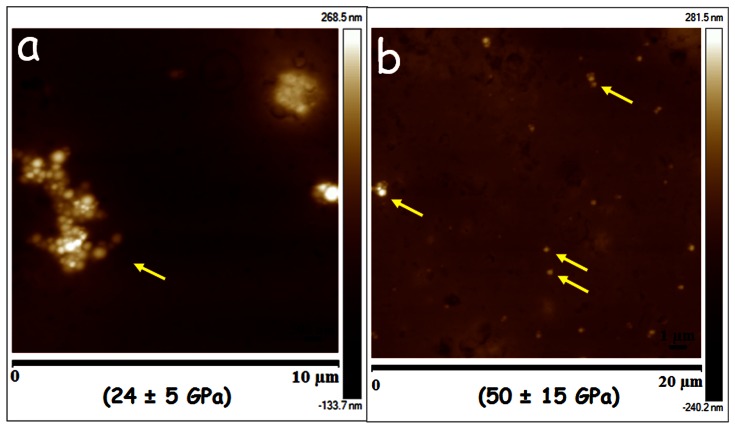
Tissue sections (15 µm) of (a) kidney and (b) liver spotted with 5 µl of CsA-NPs. The numbers in parenthesis represents tissue stiffness (representative nanoparticles are marked by yellow arrows).

RBCs were stacked and donut shaped with a stiffness of 1.6±0.4 GPa (Figure 1b and Figure 2). On spiking the CsA-NPs (100 µl) to the blood (9±3 GPa), plasma (6±2 GPa) or serum (2±1 GPa), the particles were clearly visible in all three components and their stiffness also increased significantly (Figure 1c–g and Figure 2). It should be noted that we did not expect the stiffness of the control NPs and untreated blood/plasma/serum to sum to the stiffness of blood/plasma/serum spiked with NPs. This is on account of the variance in the precise particle number, and which cannot be equally exactly controlled since dosing of NPs must be by volume of suspended particles. The consequence of an increase in the stiffness of NPs and the treated RBCs may be damaging levels of shear stress on endothelial layer, which could be considered analogous to sickle cell anaemia. [23] With regard to kidney sections, the control (untreated) tissue (Figure 1h) was much softer compared to the tissue treated with the CsA-NPs, and the individual CsA-NPs were clearly imaged (Figure 1i, Figure 2). To further establish the role of the NPs in modulating tissue stiffness, a drop (5 µl) of CsA-NPs was added to 15 µm thick sections of kidney (2.6±0.3 GPa) and liver (7±2 GPa), resulting in a significant increase in tissue stiffness (24±5 GPa & 50±15 GPa respectively) (Figure 3 a & b) compared to particle treated tissue which was sectioned later and imaged (Figure 1i). This clearly establishes the role of particle distribution in the tissues and their concentration in altering tissue stiffness. The *ex vivo* studies established the experimental platform for the subsequent *in vivo* experiments where the challenge was to track the CsA-NPs distribution after *iv* or *po* administration.

### In vivo Studies

**Figure 4:**
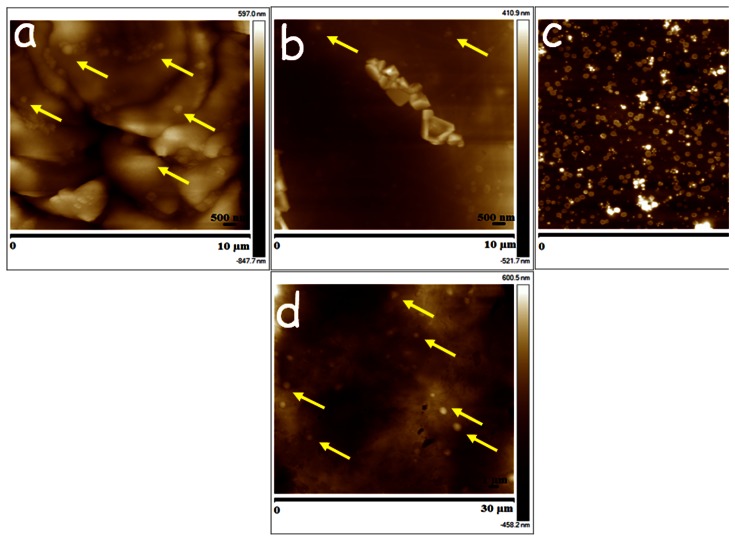
Tissue sections (a) blood, (b) plasma, (c) serum and (d) liver of rats which were dosed *iv* with 15 mg/Kg CsA-NPs and sacrificed 15 min post-dosing (representative nanoparticles are marked by yellow arrows).

The AFM images revealed CsA-NPs distribution in various tissues within 15 min after dosing and the particle presence altering the tissue stiffness (Figure 2 & 4). Though we could not observe the particle distribution in tissues such as kidney and brain sections, their stiffness was much higher compared to the respective controls that could be due to the distribution of NPs in these tissues (*Supplement Information Figure S4*).

**Figure 5:**
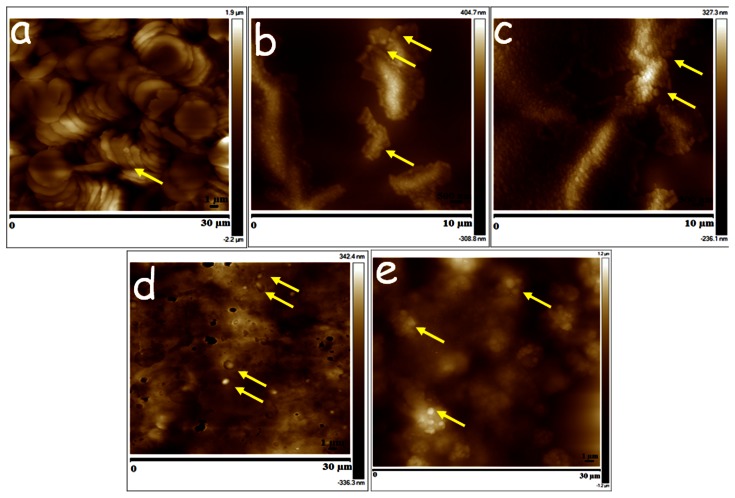
Tissue sections (a) blood, (b) plasma, (c) serum (d) liver and (e) small intestine of rats which were dosed *peroral* with 15 mg/Kg CsA-NPs and sacrificed 6 h post-dosing (representative nanoparticles are marked by yellow arrows).

On *po* dosing, the CsA-NPs were tracked into the blood after 6 h post-dosing, and were also detected in plasma and serum samples at 6 h (Figure 5 a–c). The stiffness of these samples was much higher compared to their respective controls but lower than for the *iv* group. This difference may be attributed to the amounts of NPs absorbed after *po* dose. Even in the *po* group, NPs were detected only in the liver (Figure 5d) but not the kidney and brain or blood/plasma samples of 2 and 4 h (*Supplement Information Figure S5*). The stiffness was also not as pronounced as it was in the *iv* group for the same tissues (Figure 2). This could be due to the limited absorption of the CsA-NPs within 6 h where a major portion of the particles on *po* dosing remained in the small intestine (Figure 5e) which is not surprising as majority of the reports on PLGA nanoparticles containing drugs have shown maximum blood concentrations drug profiles 24 h or beyond. [21], [24] The current study provides the first proof that label-free drug encapsulated NPs can be tracked in various tissues, though the pathways of uptake on *po* dosing remain to be investigated.

In summary, we demonstrate for the first time the presence of label-free, drug-encapsulated NPs in the tissues and the concomitant change in the local mechanical properties of the tissue (*Supplement Information Figure S6*). The AFM methodology proposed here now allows us to relate the clinical outcome post-dosing of an animal with a nanomedicine’s morphological properties of size, shape, topology, etc. [25] Given the imaging resolution of AFM it could further be envisaged that NP degradation *in vivo* could also be related to the tissue mechanical properties. This would allow us to discriminate between toxicity caused by drug *versus* particle and drug-encapsulated particle. The ability to detect NPs in blood also envisages a simple *in vitro* diagnostic tool for monitoring post-dosing without animal sacrifice. The present study holds importance in the context of unconventional drug delivery technologies, such as nanoparticles, that are currently exploited in the industry for product life cycle extension [26].

## Supporting Information

Figure S1 **Particle size measured by zeta sizer.**
(DOC)

Figure S2 **Particle size measured by nanoSight.**
(DOC)

Figure S3 **Schematic of experimental set-up and outputs.**
(DOC)

Figure S4 **Intravenous treatment group.**
(DOC)

Figure S5 **Oral treatment group.**
(DOC)

Figure S6 **Nanoparticle presence in tissue increase the stiffness, will this tissue stiffness help understand nanotoxicology?**
(DOC)
