# Supplementary material for: Atomic Force Microscopy Images Label-Free, Drug Encapsulated Nanoparticles In Vivo and Detects Difference in Tissue Mechanical Properties of Treated and Untreated: A Tip for Nanotoxicology
Source: PLoS One. 2013 May 28;8(5):e64490. doi: 10.1371/journal.pone.0064490 (PMC3665792; doi:10.1371/journal.pone.0064490)
Supplement: Figure S1 — Particle size measured by zeta sizer. (DOC) [file pone.0064490.s001.doc]

**Particle size analysis using Zeta-sizer**

Procedure - The size of nanoparticles was determined by means of dynamic light scattering (DLS) technique (Nano ZS, Malvern Instruments, Malvern, UK), taking the average of 5 measurements. The polydispersity index (PDI) which is a dimensionless number indicating the width of the size distribution, having a value between 0 and 1 (0 being for monodispersed particles) was also obtained. Results: 280 ± 15 nm and PDI is 0.078 ± 0.006

Figure S1
