# Supplementary material for: Atomic Force Microscopy Images Label-Free, Drug Encapsulated Nanoparticles In Vivo and Detects Difference in Tissue Mechanical Properties of Treated and Untreated: A Tip for Nanotoxicology
Source: PLoS One. 2013 May 28;8(5):e64490. doi: 10.1371/journal.pone.0064490 (PMC3665792; doi:10.1371/journal.pone.0064490)
Supplement: Figure S2 — Particle size measured by nanoSight. (DOC) [file pone.0064490.s002.doc]

**Nanoparticle Tracking Analysis (NTA)**

Procedure - Nanodroplet size distribution and estimated concentrations were determined with a Nanosight LM10 instrument with temperature control unit. Nanosight NTA2.1 software was used to analyze videos and calculate the size and concentration of nanodroplets. The camera setting of the instrument was set using the ‘Autosettings’ option on the software to allow the software to optimize the shutter and gain settings. The sample was introduced into the viewing unit, and an image of the particles’ scattering of the laser light was captured by a CCD camera attached to a microscope. A video of the sample was recorded and processed, with each observed individual particle ‘tracked’ by the nanoparticle tracking analysis (NTA) software. Each video was recorded for 60s and the processing parameters of brightness and gain were optimized by the software. From the Brownian motion analysis the particle size was calculated. The diffusion coefficient from the mean squared displacement of the particle tracked was calculated, and substituted into the Stokes-Einstein equation to obtain the particle hydrodynamic diameter. Estimation of the particle concentration was based on the particle count in the illuminated volume calculated from the dimensions of the field of view (at a given magnification) and the dimension of the laser beam. The particle concentration measurements are subject to a variation of up to 25% between identical samples. Particle size 254 ± 72 nm

Figure S2
