# Supplementary material for: Atomic Force Microscopy Images Label-Free, Drug Encapsulated Nanoparticles In Vivo and Detects Difference in Tissue Mechanical Properties of Treated and Untreated: A Tip for Nanotoxicology
Source: PLoS One. 2013 May 28;8(5):e64490. doi: 10.1371/journal.pone.0064490 (PMC3665792; doi:10.1371/journal.pone.0064490)
Supplement: Figure S3 — Schematic of experimental set-up and outputs. (DOC) [file pone.0064490.s003.doc]

**Details of QNM**

The QNM method is based on peak force tapping technology, during which the cantilever is oscillated in a similar way as in tapping mode, and also in each time the tip and the sample are brought together a force curve is captured (similar way as on force versus distance mode). Therefore, the method produce an image with forces and then the system (with the help of the NanoScope Analysis software) determines a DMT (Young’s) modulus using the Derjaguin-Muller-Toporov (DMT) equation and finally produce a DMT modulus image (Figure) where from there we can extract the Young’s modulus values.


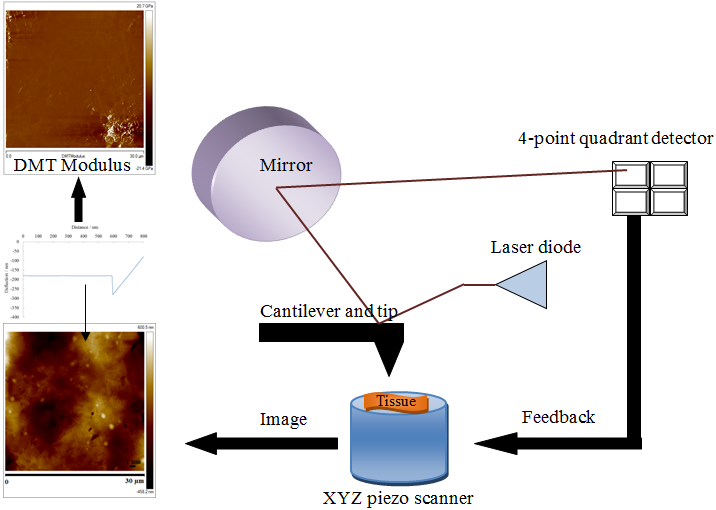


Figure S3
