# Supplementary figures and images for: Atomic Force Microscopy Images Label-Free, Drug Encapsulated Nanoparticles In Vivo and Detects Difference in Tissue Mechanical Properties of Treated and Untreated: A Tip for Nanotoxicology
Source: PLoS One. 2013 May 28;8(5):e64490. doi: 10.1371/journal.pone.0064490 (PMC3665792; doi:10.1371/journal.pone.0064490)

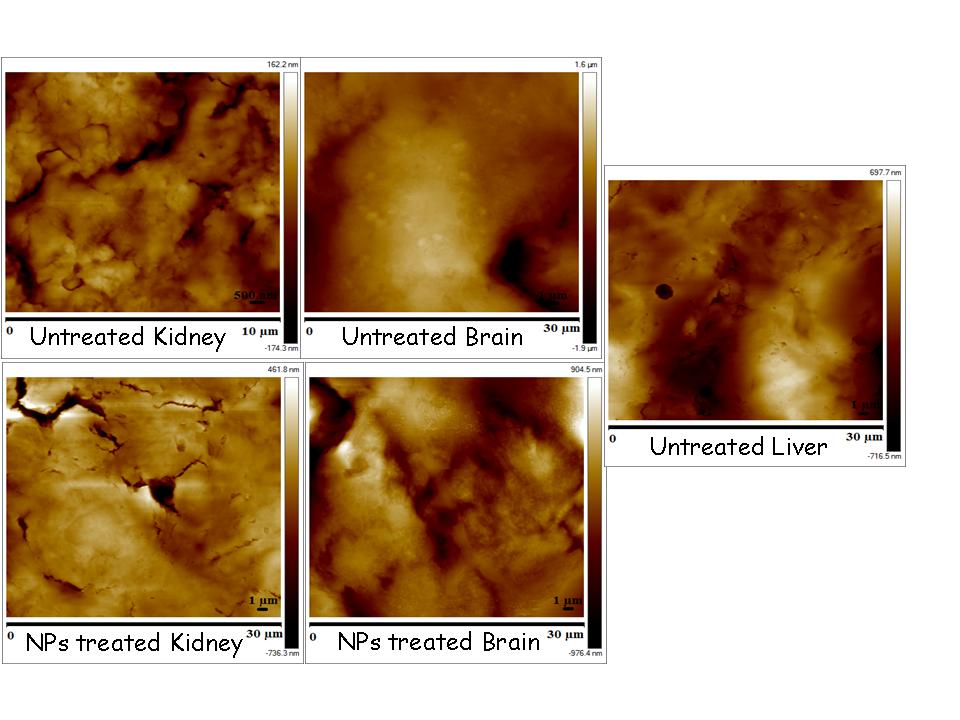


Figure S4

Supplement: Figure S4 — Intravenous treatment group. (DOC) [file pone.0064490.s004.doc]

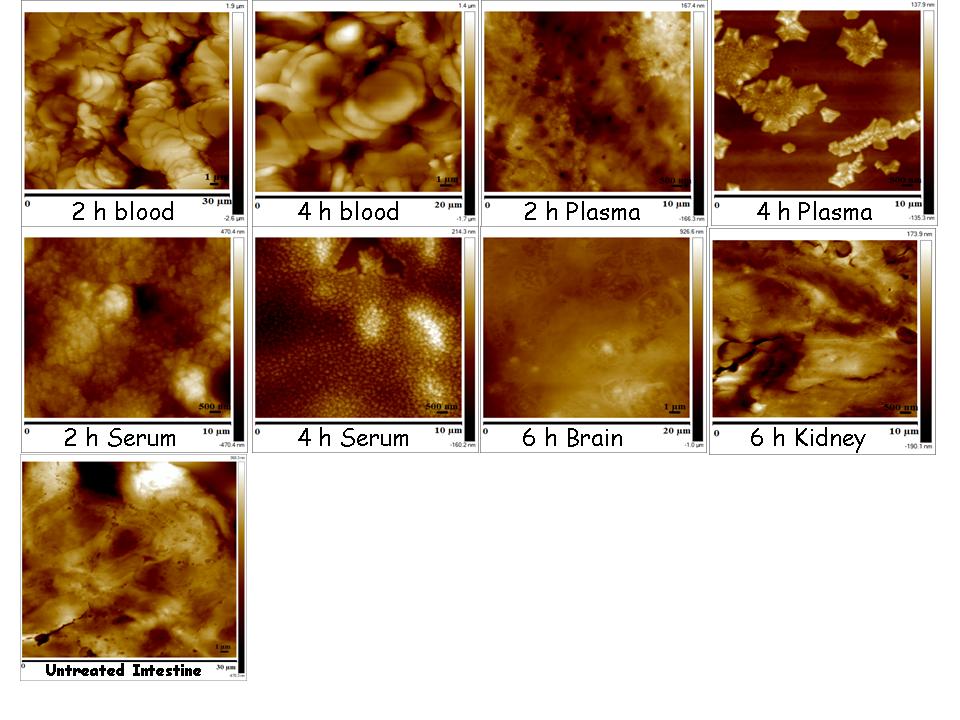


Figure S5

Supplement: Figure S5 — Oral treatment group. (DOC) [file pone.0064490.s005.doc]

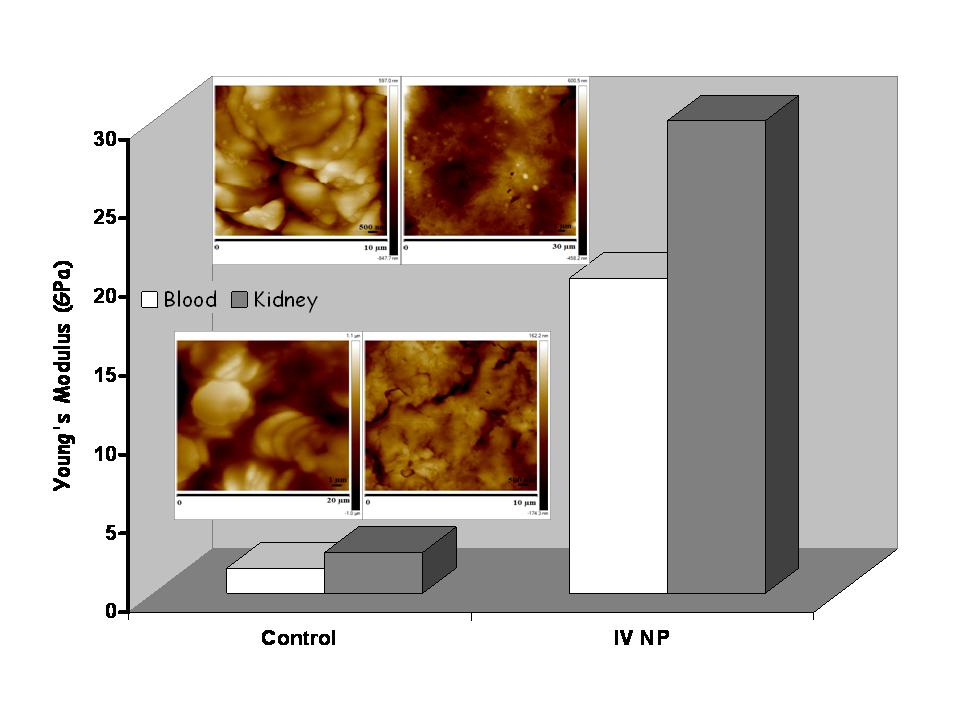
 Figure S6

Supplement: Figure S6 — Nanoparticle presence in tissue increase the stiffness, will this tissue stiffness help understand nanotoxicology? (DOC) [file pone.0064490.s006.doc]
